# A Methodological Framework for AI-Assisted Diagnosis of Ovarian Masses Using CT and MR Imaging

**DOI:** 10.3390/jpm15020076

**Published:** 2025-02-19

**Authors:** Pratik Adusumilli, Nishant Ravikumar, Geoff Hall, Andrew F. Scarsbrook

**Affiliations:** 1 Department of Clinical Radiology, Leeds Teaching Hospitals NHS Trust, Leeds LS9 7TF, UK; 2 Leeds Institute of Medical Research, University of Leeds, Leeds LS2 9NL, UK; 3 School of Computer Science, University of Leeds, Leeds LS2 9JT, UK; 4 Department of Medical Oncology, Leeds Teaching Hospitals NHS Trust, Leeds LS2 9JT, UK; 5 Leeds Institute for Data Analytics, University of Leeds, Leeds LS2 9NL, UK

**Keywords:** ovarian cancer, deep learning, CT imaging, MRI, artificial intelligence, multiple-instance learning, transformer-based models

## Abstract

**Background:** Ovarian cancer encompasses a diverse range of neoplasms originating in the ovaries, fallopian tubes, and peritoneum. Despite being one of the commonest gynaecological malignancies, there are no validated screening strategies for early detection. A diagnosis typically relies on imaging, biomarkers, and multidisciplinary team discussions. The accurate interpretation of CTs and MRIs may be challenging, especially in borderline cases. This study proposes a methodological pipeline to develop and evaluate deep learning (DL) models that can assist in classifying ovarian masses from CT and MRI data, potentially improving diagnostic confidence and patient outcomes. **Methods:** A multi-institutional retrospective dataset was compiled, supplemented by external data from the Cancer Genome Atlas. Two classification workflows were examined: (1) whole-volume input and (2) lesion-focused region of interest. Multiple DL architectures, including ResNet, DenseNet, transformer-based UNeST, and Attention Multiple-Instance Learning (MIL), were implemented within the PyTorch-based MONAI framework. The class imbalance was mitigated using focal loss, oversampling, and dynamic class weighting. The hyperparameters were optimised with Optuna, and balanced accuracy was the primary metric. **Results:** For a preliminary dataset, the proposed framework demonstrated feasibility for the multi-class classification of ovarian masses. The initial experiments highlighted the potential of transformers and MIL for identifying the relevant imaging features. **Conclusions:** A reproducible methodological pipeline for DL-based ovarian mass classification using CT and MRI scans has been established. Future work will leverage a multi-institutional dataset to refine these models, aiming to enhance clinical workflows and improve patient outcomes.

## 1. Introduction

Ovarian cancer (OC) is a heterogeneous group of neoplasms originating in the ovaries, fallopian tubes, and peritoneum. The commonest histological subtype is epithelial ovarian carcinoma, but there are extensive morphological variations within the disease spectrum, including benign, borderline, and malignant lesions. OC is the seventh most common female malignancy and a leading cause of death from gynaecological cancer worldwide, accounting for 5% of all female cancer deaths and 3% of overall cancer deaths [1,2,3].

There are no validated screening strategies for the early detection of ovarian cancer. In 2016, a UK randomised controlled trial (RCT) consisting of 202,000 women concluded that there was insufficient evidence to recommend screening [4]. The lack of effective screening combined with an often late clinical presentation with non-specific symptoms, such as abdominal pain, bloating, and fatigue, leads to a high rate of morbidity and mortality. In the UK, roughly 70% of patients present with advanced disease and distant metastases [1].

The assessment of ovarian masses largely relies on imaging, biomarker measurements, and multidisciplinary team (MDT) discussion. Imaging is fundamental for ovarian cancer diagnosis and management, helping to distinguish benign adnexal lesions from potential malignancies and identifying metastatic disease.

Ultrasonography is the primary imaging modality for the initial evaluation of ovarian masses. A transvaginal ultrasound is an accessible, real-time modality that provides detailed visualisation of ovarian lesions. The International Ovarian Tumor Analysis (IOTA) criteria may be used to classify masses based on their morphological features, blood flow, and simple sonographic rules [5]. These rules allow for the confident identification of approximately 75% of adnexal masses, with a sensitivity of around 90% and a specificity of around 95% [6]. Inconclusive and malignant cases warrant further evaluation. The Risk of Malignancy Index (RMI) was introduced as a scoring system combining ultrasound findings, serum CA-125 levels, and menopausal status, and is widely used in clinical practice to quantify the likelihood that an adnexal mass is malignant [7]. An RMI above 200 prompts a referral to a gynaecologic oncology specialist multidisciplinary team for further care. Using such an ultrasound-based initial triage helps ensure that women with likely benign masses are monitored or managed conservatively, whereas those with inconclusive or high-risk features are promptly directed to appropriate further investigations and specialist management.

In cases where the ultrasound findings are indeterminate or suggestive of a malignancy, computed tomography (CT) and magnetic resonance imaging (MRI) are the subsequent imaging modalities of choice. A CT of the abdomen and pelvis allows for the assessment of disease extent and an MRI, whilst not routinely used for initial assessments, is a valuable problem-solving tool for characterizing masses due to its superior soft-tissue contrast.

Whilst a histopathological diagnosis is the gold standard for confirming a malignancy, the point at which a biopsy is obtained may vary (Supplemental Figure S1) [8]. If a suspected malignant lesion is deemed resectable based on the imaging findings, the patient may go straight to surgery, and tissue diagnosis is established intraoperatively. If the patient is not suitable for surgery, if there is any doubt about operability, or if neoadjuvant chemotherapy may be suitable, a tissue diagnosis is used to confirm the malignancy and subtype. Therefore, the imaging findings and blood results may heavily direct the decision to proceed with a primary cytoreductive surgery.

The interpretation of imaging can be challenging and somewhat dependent on the expertise of the radiologist. This is especially true in borderline cases where the findings may be ambiguous, leading to dilemmas during MDT meetings, where decisions about further management, such as the type and extent of surgery, rely heavily on accurate imaging interpretation.

The current treatment options include surgery, radiotherapy, and chemotherapy, which have seen only slight improvements in patient survival rates over the years. Surgery risks complications, which can be mitigated in some patients, particularly in those with low-risk/benign lesions where an operation can be avoided. Therefore, a more accurate method of lesion classification to guide surgical decision-making could significantly improve patient outcomes.

Advancements in artificial intelligence (AI) and deep learning (DL) are ushering in a new era of medical diagnostics. These cutting-edge technologies have shown considerable promise for enhancing diagnostic accuracy across various healthcare domains, particularly in imaging [9,10,11]. The ability to process vast amounts of data and identify intricate patterns mean that these techniques could significantly aid in the accurate classification of ovarian lesions [11,12,13,14]. This potential enhancement in diagnostics could provide clinicians with invaluable insights to make informed treatment decisions. However, it is important to note that these technologies are still undergoing exploration and research, and their transition to clinical use necessitates further validation.

The aim of this study was to develop a methodological pipeline to evaluate AI-assisted diagnosis and phenotyping of ovarian masses using CT and MR imaging data routinely acquired for staging or detailed lesion assessment. A growing body of literature has demonstrated the potential of radiomics in ultrasounds for ovarian lesion classification [12]. In this paper, however, we focus on CT and MRI, since these modalities are routinely used for staging or further characterisation when the ultrasound findings are equivocal. The methodological framework will be used to develop and train DL models by utilising the pre-treatment imaging data to differentiate between benign, malignant, and borderline ovarian lesions. Two methodological approaches have been designed, incorporating both classification without segmentation and classification with a 3D region of interest (ROI) bounding box around the primary lesion. Pipelines utilising both traditional convolutional neural network (CNN)-based architectures and models available through the Medical Open Network for Artificial Intelligence (MONAI) have been developed. The primary goal was to establish a reproducible and clinically relevant process for training and evaluating DL models. The integration of an AI-assisted clinical decision support system providing enhanced imaging interpretations may help guide optimal treatment decision-making.

## 2. Materials and Methods

### 2.1. Ethical Approval

Formal approval for the use of real-world patient data in this study was granted by the UK Integrated Research Application System (IRAS), reference number 277122 (RCD-Onc: Enhancing understanding and prediction of cancer outcomes with baseline characteristics from routinely collected data) [15]. This approval was conferred on 3 December 2019 and permitted both retrospective and prospective data usage. An additional institutional data access committee approval (Ref: LTH22020 RCD-Onc) ensured that any patients who opted out of research via the UK National Data Opt-Out service [16] were excluded from the dataset prior to analysis. Under this National Data Opt-Out policy, secondary use of anonymised patient data is permitted (e.g., research and planning) unless they explicitly opt out. Therefore, no individual signed consent forms exist for this study. All relevant approvals and governance are in place via IRAS/HRA approval and through adherence to the National Data Opt-Out policy.

### 2.2. Patient Selection

**Figure 1:**
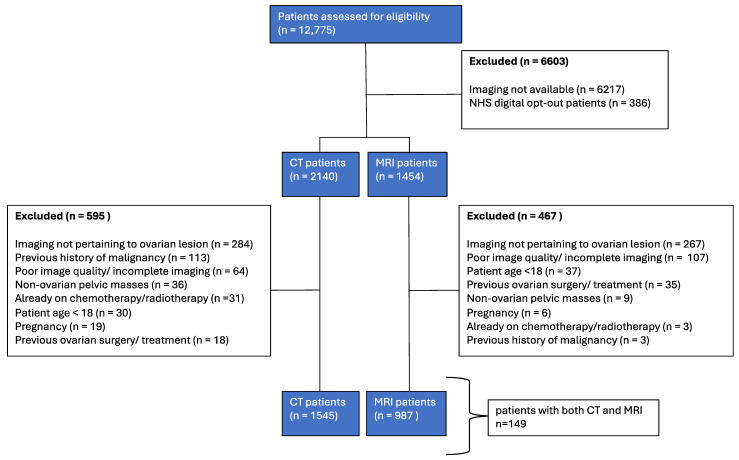
Patient selection flow diagram.

Internal cohort patient selection is illustrated in Figure 1. Patient characteristics are detailed in Table 1 and Table 2. Patient characteristics for the external validation cohort are detailed in Table 3 and Table 4.

Cohort 1: patients undergoing CT and/or MRI for investigation of ovarian masses between January 2005 and December 2021 were identified retrospectively from a single institution (Leeds Teaching Hospitals NHS Trust).

Cohort 2: data for external validation were retrospectively identified from patients undergoing CT and/or MRI for investigation of ovarian masses at multiple regional hospitals between January 2005 and December 2021.

Cohort 3: a separate external validation CT data cohort was obtained from the Cancer Genome Atlas (TGCA-OV dataset).

Gold standard or “ground truth” pathology diagnoses for all patients, including controls, were corroborated using the final histopathological report. Imaging findings were validated by subspecialty Consultant Radiologist radiology reports and specialist Gynaecology MDT discussion notes available from the regional electronic patient records (PPM+, Leeds, UK).

### 2.3. Exclusion Criteria

Patients were excluded from this study if imaging was of poor quality or incomplete. For CTs, this included artefacts and incomplete (truncated) scan data. For MRIs, this included motion artefacts, scans with incomplete pelvic region coverage, or instances where only part of the scan volume was covered in a single pass. Patients who had already undergone ovarian surgery or commenced chemotherapy or radiotherapy prior to imaging were excluded.

### 2.4. Inclusion Criteria

Patients were required to have pre-operative imaging available.

Imaging data for selected patients were extracted from the institutional PACS (Picture Archiving and Communication System) (Enterprise Imaging, AGFA Healthcare, Belgium).

### 2.5. Selection of Representative Sample for Model Development

A total of 500 CT scans and 200 MRI scans were chosen for the preliminary dataset for model development. The proportion of each diagnosis was kept at the same ratio as that of the full dataset to ensure accurate representation, as well as to allow for testing of class imbalance compensation methodologies.

### 2.6. Imaging Protocol

#### 2.6.1. CT

Portal venous phase contrast-enhanced CT scans of the abdomen and pelvis were acquired using multiple scanners over the course of this study (Table 5 and Table 6). All images were reconstructed with manufacturer-specific iterative algorithms.

#### 2.6.2. MRI

Pelvic MRI studies were obtained using multiple scanners over the course of this study (Table 5 and Table 6). T2-weighted, T1-weighted, fat-saturated T1-weighted, and diffusion-weighted imaging (DWI) sequences were utilised.

### 2.7. Model Implementation

All code was written in Python 3.9.

### 2.8. Image Extraction and Preprocessing

**Figure 2:**
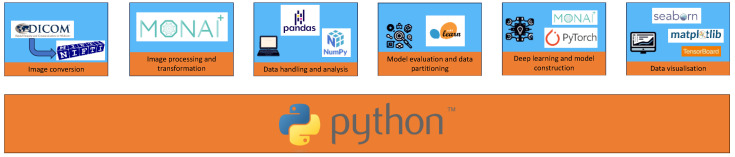
Model development process.

CT and MRI scans were preprocessed to ensure imaging data standardisation prior to analysis. The workflow was categorised into distinct stages (Figure 2).

CT and MRI scans were exported from PACS in Digital Imaging and Communications in Medicine (DICOM) format. Although DICOM is the standard format for storing and transmitting medical imaging data, it is not ideal for ML and DL tasks for several reasons.

DICOM contains detailed metadata, including sensitive patient information, study details, and acquisition parameters. Additionally, DICOM divides each imaging slice into separate 2D files, creating inconsistencies in data handling. In contrast, the NIfTI (Neuroimaging Informatics Technology Initiative) format offers a streamlined approach by consolidating the data into unified 3D volumes, which simplifies data handling and subsequent integration with ML and DL libraries. NIfTI also helps improve data consistency by standardising image dimensions and voxel sizes, and absence of patient-specific information mitigates privacy concerns.

The dcm2niix (https://github.com/rordenlab/dcm2niix (v1.0.20240202), accessed date: 22 January 2025) library [17,18,19,20] was employed for DICOM to NIfTI conversion. Key metadata were preserved in a JavaScript Object Notation (JSON) sidecar file for each NIfTI file, containing key acquisition parameters, patient information, and scanner details extracted from the DICOM header. Using the “wholeBody_ct_segmentation” model from the Medical Open Network for Artificial Intelligence (MONAI) framework Model Zoo (https://monai.io/model-zoo.html (v1.4.0), accessed date: 22 January 2025) each 3D CT volume was subjected to automated sacrum segmentation. Once identified, scans were cropped to retain the sacrum along with 150 cm of cranial extension above its most superior portion. Pelvic MRI scans did not require any cropping.

With the “NiBabel” Python library (https://nipy.org/nibabel/gettingstarted.html (v3.2.2), accessed date: 22 January 2025), each imaging study was reoriented to a standard reference frame. A fixed range of intensities was applied to maximise soft-tissue contrast for CT scans. For MRI data, ComBat harmonisation was applied by adjusting the intensity distributions across different scanners to match a common reference distribution, thereby correcting for scanner-specific biases (https://github.com/Jfortin1/neuroCombat, accessed date: 22 January 2025). Each 3D scan then underwent resizing to a uniform shape and intensity normalisation to ensure consistency in input size and voxel values. The “Scikit-Learn” library (https://scikit-learn.org (v1.5.2), accessed date: 22 January 2025) was used to split data for model training, validation, and testing.

To improve generalizability and increase sample variety, data augmentation was applied. This included random transformations, such as random affine transformations, flipping, and elastic deformations to increase the variety of samples (Supplemental Table S1). Class imbalance was tackled in part with minority class oversampling. At the end of these steps, data were standardised, balanced, and augmented, and ready to be fed into a deep learning model as PyTorch tensors.

Itk-Snap (http://www.itksnap.org (v4.0.1), accessed date: 22 January 2025) was used to draw a spherical region of interest (ROI) encompassing the lesion in the 3D scan data. The segmented ROI could then be passed into the existing model architectures as the primary input for training, allowing models to focus on the most pertinent features within the specified region. The ROI could be exported in the standard Neuroimaging Informatics Technology Initiative file format (.nii), and used as an additional input for the DL networks.

**Figure 3:**
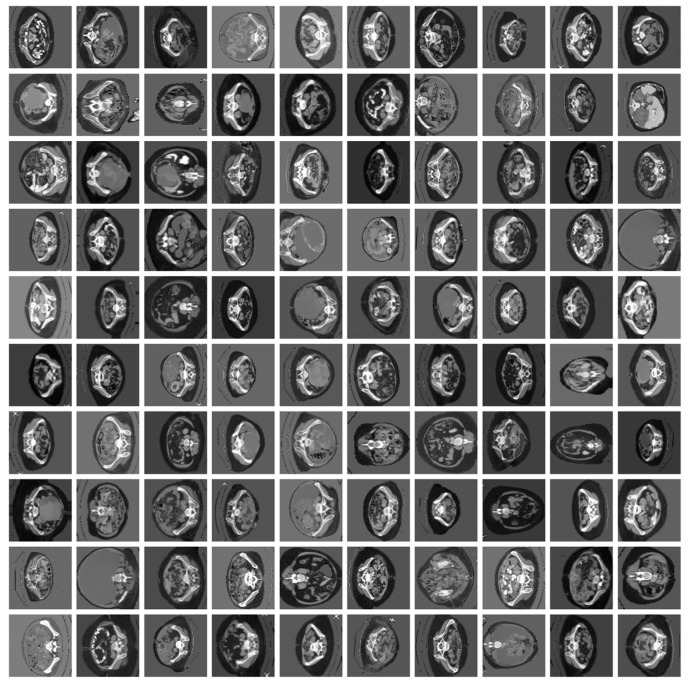
Matplotlib visualisation of input data.

**Figure 4:**
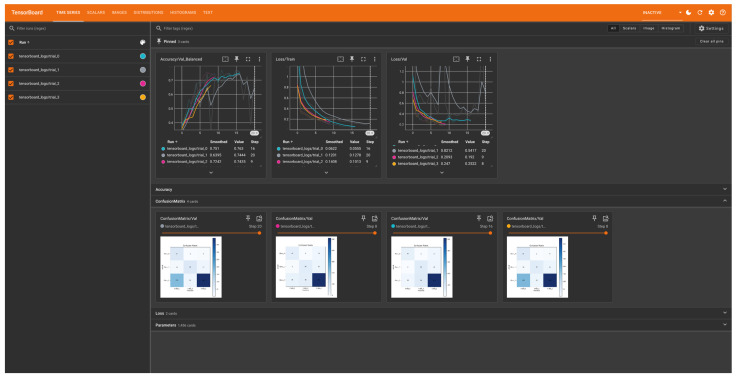
TensorBoard visualisation of the model training process, illustrating real-time monitoring of metrics and performance.

To help interpret data, the “Matplotlib” (v3.10.0) Python library was used to visualise output data (Figure 3). “TensorBoard” (https://www.tensorflow.org/tensorboard (v2.18.0), accessed date: 22 January 2025) was also employed to collect and present a range of performance metrics. “TensorBoard” facilitates tracking and comparison of different model runs, providing clear insights into training progress and results with an interactive dashboard (Figure 4).

“Pandas” (v2.2.3) and “NumPy” (v1.26.4) Python libraries were utilised to organise and analyse imaging data. “Pandas” enabled creation of structured data frames and “NumPy” was used to create data arrays.

### 2.9. Model Architectures

DL encompasses a multitude of architectures designed to handle complex image classification tasks. Radiologists are often asked to differentiate between benign, borderline, and malignant ovarian masses, a crucial, yet challenging task. Beyond this differentiation, an emerging interest is to determine if these architectures can also discriminate between pathological ovarian cancer subtypes. This level of detail could provide an additional layer of precision in diagnosis and management.

All models were implemented using the PyTorch-based (v2.5.1+cu124) MONAI (v1.4.0) framework. This provides a suite of tools optimised for handling complex healthcare data, including CT and MRI. These tools include optimised data transformation and preprocessing utilities, aside from network architectures that are well suited for working with 3D volumetric data. MONAI also hosts the Model Zoo, a repository of pre-trained models. MONAI provides a strong foundation for both whole-volume and ROI-based analyses.

### 2.10. CNN-Based Architectures (DenseNet and ResNet)

CNNs are designed to learn spatial hierarchies of features automatically and adaptively from images. The architecture consists of an input layer, several hidden layers, and an output layer. Hidden convolutional layers employ filters to detect patterns, such as edges and shapes, through a series of convolutional, pooling, and fully connected layers. CNNs can capture hierarchical patterns in data, making them exceptionally efficient for image classification tasks.

These architectures permit extraction of a wide range of imaging features, which may increase the precision of imaging analysis. Radiological images often contain hidden features that can influence diagnostic outcomes. The layered structure of these networks is adept at understanding these hierarchies. These architectures have displayed promising performances in various prior studies [21,22,23,24,25,26,27,28,29,30,31,32], making them promising candidates for the classification of ovarian masses.

### 2.11. DenseNet

DenseNets are a variation of CNNs, utilising dense connections between layers, with each layer receiving feature maps from all preceding layers. This promotes feature reuse, improving efficiency and reducing the number of parameters (and mitigating the risk of overfitting). DenseNets can be particularly advantageous in medical imaging, where data size is often limited compared to conventional big data.

### 2.12. ResNet

ResNets introduce the concept of skip connections or shortcut connections. These connections allow a network to skip layers during forward and backward passes, addressing the vanishing gradient problem often encountered in deeper networks. ResNets enable the capture of more intricate features because of the residual blocks containing these skip connections. This can be beneficial when trying to discern nuanced differences between ovarian pathological subtypes.

### 2.13. Transformer-Enhanced Architecture—UNeST (Universal Network with Nested Transformer)

UneST architecture is built on the foundation of UNet architecture. Traditional UNet architectures employ a U-shaped structure with a contracting path to capture features and an expanding path to achieve localisation. UNeST architecture enhances this by integrating nested transformer layers, adding powerful attention mechanisms to a network. The transformer layers can model long-range dependencies, allowing the architecture to understand global relationships within an image. This results in a robust solution for detail localisation and global context.

### 2.14. Multiple-Instance Learning (MIL)

DL models are typically trained using single-instance learning, where each instance in a training set is labelled. However, a single 3D volume may contain multiple distinct regions (patches), which collectively determine its label. Multiple-Instance Learning (MIL) considers a “bag” of patches with one label, allowing a model to make inferences based on collective information by focusing on specific informative regions rather than whole images. This approach can reduce computational load.

MONAI offers 2D MIL utilities; we implement a custom 3D MIL pipeline to fully leverage volumetric imaging data for ovarian mass classification. Recent advances, such as Attention MIL [33,34,35,36], have shown improved focus on relevant regions of interest for classification tasks compared to conventional MIL. Attention MIL incorporates attention layers within the MIL framework that help the model highlight and weigh the most relevant instances within a bag. This is particularly beneficial when subtle details in certain areas may significantly impact diagnosis.

### 2.15. Optimizing Hyperparameters

Hyperparameters control the learning process of an algorithm. Choosing optimal hyperparameters is critical to refining a network’s performance. “Optuna” (v4.1.0) (https://optuna.org, accessed date: 22 January 2025) is a Python hyperparameter optimisation library which can be implemented for PyTorch-based models to systematically tune parameters through an automated search process. Hyperparameters include batch_size (the number of samples processed simultaneously in each training step), learning rate (how aggressively the model updates its weights), and weight decay (a factor that helps control overfitting).

### 2.16. Handling Class Imbalance

As previously described, minority class oversampling was used to ensure adequate exposure during training. Focal loss from MONAI was also employed, which leverages two key parameters, alpha to prioritise minority classes and gamma to emphasise hard-to-classify samples. A dynamic class weighting strategy was applied after each training epoch, where “minority_acc_threshold” checked if there were any class lags in accuracy, and “minority_boost_factor” magnified the weight of that class in the loss function, which effectively penalised the model for misclassifying minority classes.

### 2.17. Classification Tasks

Two classification tasks were evaluated using both CT and MRI data, with and without including the ROI:

1. Three-way classification to differentiate between benign, borderline, and malignant lesions.
2. Four-way classification of lesions into one of the following histological subtypes: benign, high-grade serous, other epithelial, and non-epithelial.

### 2.18. Model Overview

Please refer to Supplemental Table S2 for a list of software tools and Supplemental Table S3 for a layer-by-layer breakdown of the proposed models.

### 2.19. ResNet

#### Model Architecture

**Figure 5:**
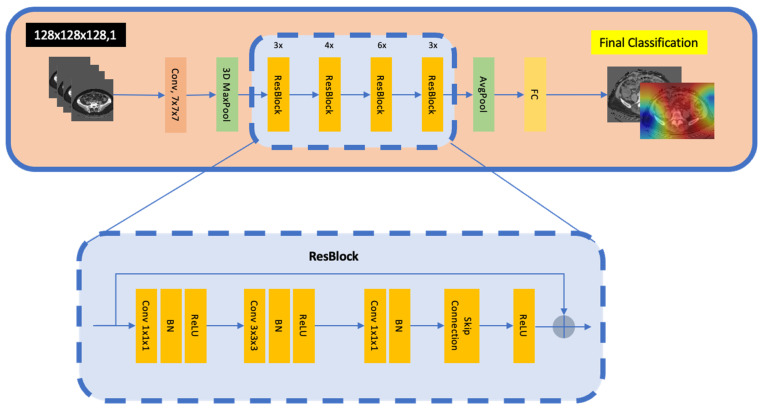
Schematic of the 3D ResNet architecture. The pipeline begins with a Conv 7 × 7 and a 3D MaxPool operation to reduce the spatial resolution, followed by multiple ResBlocks (each comprising 1 × 1 and 3 × 3 × 3 convolutions, batch normalisation, ReLU activations, and skip connections). A global AvgPool layer then condenses the volumetric features into a single vector, which is passed to an FC layer for final classification. The inset illustrates the typical ResBlock layout with Conv–BN–ReLU sequences, skip connections, and a final post-addition ReLU. Conv: convolution; BN: batch normalisation; ReLU: Rectified Linear Unit; AvgPool: global average pooling; FC: fully connected.

Figure 5 illustrates the 3D ResNet architecture. It begins with convolutional blocks, each comprising a Conv3D layer followed by batch normalisation and a Rectified Linear Unit (ReLU) activation function, which work together to identify and transform spatial features in the data.

The architecture then progresses to encoder blocks, which consist of a convolutional block with a MaxPooling3D layer. This reduces the size of the feature maps, simplifies the data, and prepares them for deeper layers. This structure allows for the extraction of increasingly complex features at varying resolutions.

After passing through a series of encoder blocks, the data flow through one final convolutional block before reaching the GlobalAveragePooling3D layer, which condenses all the spatial information into a single flattened vector. The data then pass through a dense layer, culminating in another dense layer that generates the final classification probabilities using softmax activation. The softmax function converts raw output into probabilities for each class, allowing the model to make predictions based on the class with the highest probability.

### 2.20. DenseNet

#### Model Architecture

**Figure 6:**
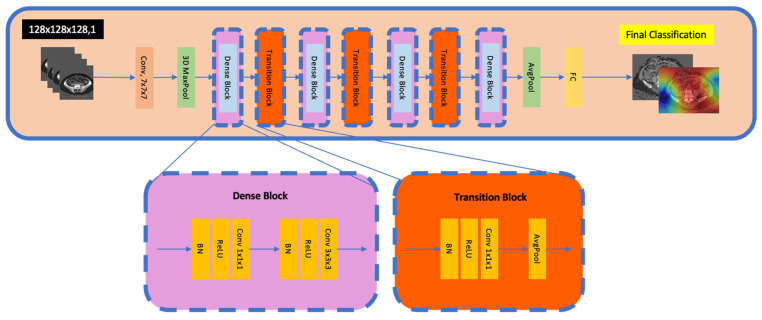
Schematic of 3D DenseNet architecture. The pipeline begins with a Conv 7 × 7 for initial feature extraction, followed by a 3D MaxPool to reduce spatial resolution. A series of dense blocks (purple) and transition blocks (orange) then iteratively expand and reduce the feature dimensions. Each dense block comprises successive Conv–BN–ReLU operations, concatenating outputs from all preceding layers, while each transition block (Conv 1 × 1 + AvgPool) reduces feature map size and channels. A global AvgPool layer aggregates the final volumetric features into a single vector, which is passed to a fully connected (FC) layer for classification. The insets illustrate the typical internal layout of a dense block and transition block.

Figure 6 illustrates the 3D DenseNet. The convolutional base layer begins with a Conv3D layer, which extracts preliminary spatial features. This is followed by batch normalisation and an ReLU activation function. A MaxPooling3D layer then downsamples the feature maps.

The core of DenseNet includes a sequence of dense and transition blocks. Dense blocks consist of multiple convolutional layers, where every new layer receives the concatenated output of all the preceding layers, ensuring direct passage of gradients during training. Transition blocks are used between dense blocks to reduce spatial dimensions and channel depth; they comprise a batch normalisation layer, a Conv3D layer, and an average pooling layer.

Transition blocks are used between dense blocks to reduce spatial dimensions and channel depth. Dense blocks comprise a batch normalisation layer, a Conv3D layer, and an average pooling layer.

After all the dense and transition blocks are complete, the model employs a batch normalisation layer, ReLU activation, and a GlobalAveragePooling3D layer to generate a flattened feature vector. DenseNet concludes with a dense layer producing the classification probabilities using softmax activation.

### 2.21. UNeST

**Figure 7:**
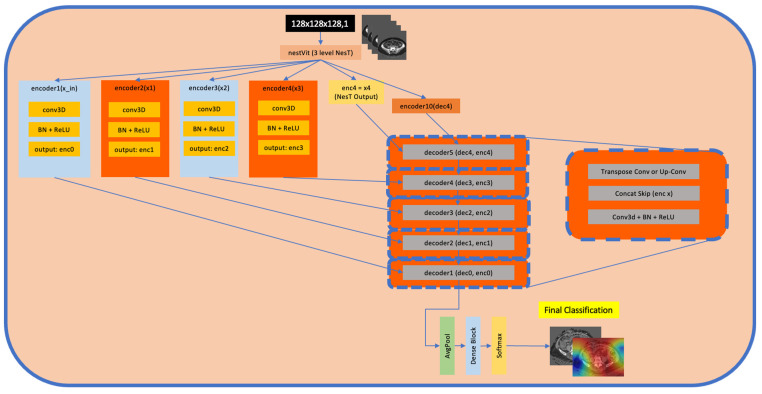
Schematic of 3D UNesT architecture. The 3D input volume is passed through a 3-level NesT to produce multi-scale feature maps {x1,x2,x3,x4}{x1,x2,x3,x4}, as well as a final feature map, xx. Each intermediate NesT output is fed into a corresponding encoder block (encoder1–encoder4encoder1–encoder4), comprising 3D convolutions (with BN/ReLU) and/or upsampling, yielding encoded features enc0–enc3enc0–enc3. The final NesT output, xx, is further processed by an additional encoder (e.g., encoder10) to expand feature depth. In the decoder phase, skip connections fuse the encoder outputs with transposed convolution layers (decoder5–decoder1) to reconstruct intermediate 3D representations. A classification head (global average pooling followed by a dense layer and softmax) aggregates the final 3D representation to produce class probabilities. NesT: nested transformer; BN: batch normalisation; dec: decoder; enc: encoder; ReLU: Rectified Linear Unit; AvgPool: global average pooling.

Figure 7 illustrates the 3D UNeST classifier model. The architecture consists of a U-Net backbone, which begins with several convolutional layers and skip connections. A GlobalAveragePooling3D layer condenses the spatial information into a single feature vector, effectively reducing the dimensionality.

This vector then flows through a classification head that consists of a sequence of fully connected layers. These utilise dropout and ReLU activation for regularisation and non-linear transformation. Ultimately, these culminate in a dense layer with softmax activation to produce the classification probabilities.

### 2.22. Attention MIL

**Figure 8:**
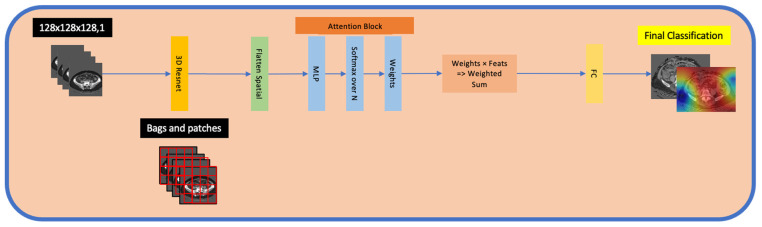
Schematic of the 3D attention-based Multiple-Instance Learning (MIL) pipeline. The pipeline starts with a 3D input volume (CT or MRI) split into multiple patches (forming a “bag”). Each patch is processed by a 3D ResNet3D ResNet-50 (Residual Network) to extract the patch-level feature embedding, which is then flattened spatially. These embeddings are passed through an attention block comprising an MLP (Multi-Layer Perceptron) and a softmax over NN patches to yield the attention weights. The weighted sum of the patch embeddings is computed according to these attention weights, producing a single aggregated feature vector. Finally, this aggregated representation is fed into an FC (fully connected) layer for the final classification. 3D: three-Dimensional; ResNet: Residual Network; MLP: Multi-Layer Perceptron; FC: fully connected.

Figure 8 demonstrates the Attention MIL classifier. In this model, data are organised into multiple bags. This structure enables the model to focus on the most relevant regions within an image. Each bag is assigned a single label that reflects the overall characteristic of the image. Within each bag, there are multiple patches, which are localised segments of the original image. An attention mechanism is employed in each of these patches to allow the model to focus on the most informative regions within each image.

The classifier begins by extracting features from each 3D patch in a bag using a Conv3D layer with ReLU activation, followed by adaptive average pooling. The pooling layer ensures that every patch in the bag has a standardised representation. Next, the attention-weighted patches are aggregated to form a single vector that represents the entire bag by multiplying each patch’s feature vectors by its corresponding attention weight. This allows the model to identify the most pertinent patches while minimizing the influence of less relevant patches. The aggregated vector then flows into a classification layer, which uses dense, fully connected layers with softmax activation to produce class probabilities. The classification label is output for the entire bag, making it effective for weakly supervised whole-image classification tasks.

### 2.23. Model Training

Training is executed over up to 100 epochs (complete cycles through the training dataset) and different batch sizes (number of training samples processed together). The total number of epochs are determined by the early stopping mechanism, which stops the training if additional cycles are not causing an improvement in the loss function. During each epoch, performance metrics are logged. Monitoring and logging are crucial for in-depth analysis and understanding of model performance, enabling identification of trends and potential areas for improvement. Optuna is used to systematically explore the hyperparameter search space to pinpoint the most promising combinations for optimal performance (Supplemental Table S4).

### 2.24. Model Compilation

Loss Function: Each model employs focal loss as the loss function. This choice is effective for tasks where there is class imbalance.

Optimiser: The Adaptive Moment Estimation (ADAM) optimiser is used across all three models. Initiated with a learning rate based on optimal hyperparameter settings, the ADAM optimiser has adaptability and effectiveness at complex model training. It adjusts learning rate dynamically, facilitating efficient convergence to optimal solutions.

Performance Metric: For all models, balanced accuracy is chosen as the primary evaluative metric. This metric quantifies average accuracy of each individual class rather than simply weighting them by frequency.

### 2.25. Callbacks and Learning Rate Scheduling

#### 2.25.1. Model Checkpointing

The model weights are saved in PyTorch Tensors (pt) format after each epoch if there is an improvement in validation performance to preserve the best state of the model. The pt file can also be used for model inference or for future fine-tuning of the model.

#### 2.25.2. Early Stopping

To prevent overfitting and to ensure training efficiency, EarlyStopping is employed in all three models. This callback ceases training if there is no noticeable improvement in validation loss over a set number of consecutive epochs. EarlyStopping ensures that the model does not learn the noise in the training data, thus maintaining its ability to generalise.

#### 2.25.3. Learning Rate Scheduling

An adaptive learning rate scheduler is used in each model’s training process. For the initial 10 epochs, the learning rate is kept constant, providing stability in the early stages of learning. After the 10th epoch, learning rate is reduced by half. This gradual reduction allows for more refined adjustments to the model’s learning, particularly as it starts converging towards optimal performance. The scheduler plays a key role in balancing the speed and accuracy of learning, adapting the training dynamics as the model evolves.

#### 2.25.4. Performance Metrics

Metrics are essential to assess the performance of ML and DL models. They provide quantitative insights into model behaviour, highlighting strengths and pitfalls. The metrics include the following:

1. Loss (Training and Validation)
  ○ Definition: Loss, synonymous with the cost or objective function, measures how closely model prediction aligns with actual data. A diminutive loss value indicates better alignment of prediction with true values.
  ○ Training Loss: demonstrates the loss computed on the training dataset, signifying the model’s fit to the data.
  ○ Validation Loss: Calculated using the validation dataset, it is a barometer for the model’s potential performance on unfamiliar data. An escalating validation loss juxtaposed with a diminishing training loss usually flags overfitting.
2. Accuracy (Training and Validation)
  ○ Definition: accuracy represents the fraction of predictions that are correct, making it particularly lucid for classification tasks.
    ▪ Accuracy = (Number of Correct Predictions)/(Total Predictions)
  ○ Training Accuracy: Quantifies the correct predictions made on the training set. Elevated training accuracy can be deceptive if the model is overfitting.
  ○ Validation Accuracy: denotes the correct predictions made on the validation set, hinting at probable performance on new data.
3. Balanced Accuracy (Validation)
  ○ Unlike standard accuracy, balanced accuracy is the average accuracy of each individual class rather than simply weighting them by frequency. By doing so, minority classes are afforded the same significance as majority classes, more accurately reflecting model performance across all classes, and preventing the dominant class from skewing the overall evaluation metric.
4. Confusion Matrix
  ○ Definition: This matrix is instrumental in classification for comprehending algorithmic performance, especially when multiple classes are at play. It meticulously juxtaposes predictions against true values.
    ▪ True Positive (TP): both actual and predicted classes are positive.
    ▪ True Negative (TN): both actual and predicted classes are negative.
    ▪ False Positive (FP): predictions are positive, but reality is negative.
    ▪ False Negative (FN): predictions are negative, yet reality is positive.
  ○ This matrix can be further mined to deduce metrics like precision, recall, and F1-score.

#### 2.25.5. Saving Performance Metrics

Metrics like loss and accuracy, charted over epochs, are collated into a CSV file. This facilitates retrospective analysis and discernment of the model’s training trajectory.

#### 2.25.6. Plotting Performance Graphs

Loss and accuracy graphs are plotted to visualise per epoch convergence, overfitting, and to visualise the model’s learning progression.

### 2.26. Grad-CAM (Gradient-Weighted Class Activation Mapping)

**Figure 9:**
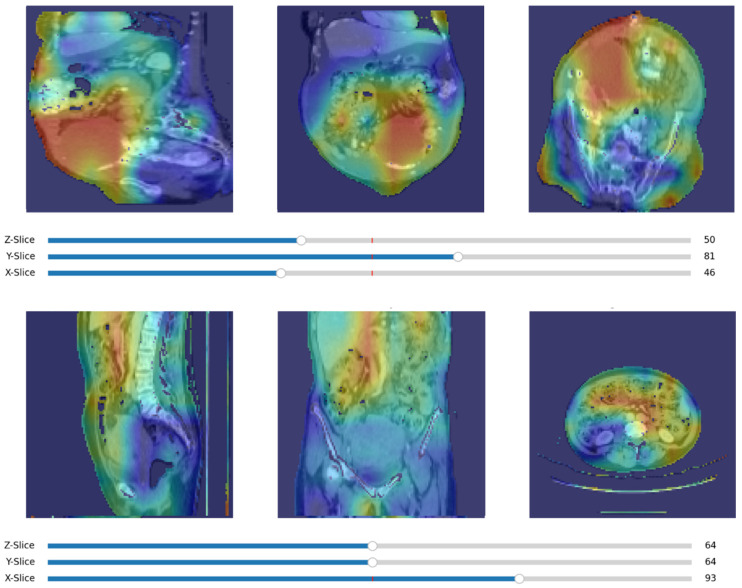
Grad-Cam visualisation: the upper image demonstrates a “hot” (red) malignant lesion and the lower image demonstrates “hot” (red) regions of omental disease.

Grad-CAM is a visualisation technique that highlights regions within an image that play a significant role in a model’s predictions. The heatmap pinpoints areas of importance and provide insights into the decision-making process of DL models. This is valuable for improving interpretability and allows end users to visualise which parts of an image contributed most to a specific prediction (Figure 9, Supplemental Video).

To generate the heatmap using Grad-CAM, first, a convolution layer is chosen. The image is processed through the model up to this chosen layer, and the feature maps are extracted. A subsequent backward pass calculates the gradient of the target prediction in relation to the feature maps, revealing how much each spatial location in the feature map contributed to the model’s output. The gradients are globally averaged for each feature map, creating a set of weights.

In summary, while metrics provide a quantitative assessment of model performance, heatmaps provide information on qualitative aspects. These visual tools aim to demystify the decision-making process of models by highlighting areas of the image that most influence the models’ predictions. Saliency maps, while invaluable for providing insights into model behaviour, come with their challenges. They can produce ambiguous or misleading visualisations, particularly for complex medical images where the distinction between relevant and irrelevant features is not always clear. Additionally, their interpretability is often contingent on the expertise of the viewer, which can vary widely among clinicians. When applied carefully, saliency maps aim to enhance our understanding and trust in AI diagnostics, offering a window into the otherwise opaque workings of deep learning models.

### 2.27. Model Evaluation and Visualisation

After completion of training, performance metrics are organised into DataFrames. These metrics are then exported as common separated values (.csv) files for future reference and in-depth analysis. This method of storage enables a comprehensive evaluation of the models’ progression throughout the training epochs, providing valuable insights into their behaviour and effectiveness. It also assists in making informed decisions regarding potential adjustments to models’ architectures or hyperparameters. Models’ architectures, along with their learned weights, are preserved in pt format. The pt file is also used for inference purposes whilst validating the models.

For testing the ROI models, the test data have a spherical ROI drawn over the lesion of interest using itk-Snap. This ensures that both the training and testing phases are focused on the specified ROI, allowing for a consistent and targeted evaluation of the models’ performance on the crucial areas of interest.

## 3. Results

A total of 1545 CT patients met the inclusion and exclusion criteria; the median age at the time of the scan was 64 years (range, 18–96 years). Among the different diagnoses, serous adenocarcinoma (29.7%, n = 459) and cysts (22.9%, n = 354) were predominant (Figure 1, Table 1).

For the MRI patient group, 987 participants met the inclusion and exclusion criteria. The median age at the time of the scan was lower than that for the CT group, at 52 years (range, 18–96 years). The MRI group predominantly consisted of patients diagnosed with cysts (53.7%, n = 530). The neoplasm benign category also had a significant representation (10%, n = 98). In contrast to the CT patients, serous adenocarcinoma constituted 5.5% (n = 54) of the MRI cohort (Figure 1, Table 2).

For the external CT dataset, 1922 participants from 55 centres met the inclusion and exclusion criteria (median age, 63; range, 18–95 years). The majority were diagnosed with a malignant disease (87.3%, n = 1679), with serous adenocarcinoma representing the largest subset (45.2%, n = 868). Borderline neoplasms accounted for 9.6% (n = 185), while benign lesions comprised 3.0% (n = 58) (Table 3).

For the external MRI dataset, 337 participants from 37 centres were included. The median age at the time of the scan was slightly lower at 55 years (range, 18–92 years). Malignant pathologies formed 48.4% (n = 163) of the diagnoses, followed by serous adenocarcinoma (17.2%, n = 58). Borderline tumours (24.9%, n = 84) and benign findings (26.7%, n = 90) made up the remainder of the cohort (Table 4).

The TGCA-OV dataset [37,38] comprised 143 CT scans from six U.S. centres, exclusively featuring cases of serous adenocarcinoma. The median patient age at the time of imaging was 61 years (range, 38–82 years).

## 4. Discussion

The goal of this study was to establish a framework for incorporating deep learning models into CT and MR imaging for ovarian mass classification. Class imbalance was addressed by employing oversampling, focal loss, and balanced accuracy, and by introducing a dynamic class weighting strategy in each training epoch. All the relevant hyperparameters, including alpha, gamma, minority_acc_threshold, and minority_boost_factor, were tuned using Optuna. Balanced accuracy was used as the final evaluation metric for accuracy given the class imbalance.

Recognising the intricacies and challenges of ovarian mass classification, the primary focus was to detail the methodology for distinguishing between benign, malignant, and borderline lesions.

To test the feasibility of developing these models, a methodological approach was undertaken using a preliminary dataset. It is essential to understand that deep learning, by its nature, often requires expansive datasets to realise its full potential. The results from this preliminary study, given the limited dataset, should be approached with caution. While they offer initial insights into the models’ structures and potentials, they may not robustly represent the models’ eventual diagnostic capacities. As a larger dataset is evaluated and the models are refined, it is anticipated that we will obtain more reflective and clinically pertinent outcomes. The provisional findings, though indicative of potential pathways, might not be definitive or sufficiently useful for broader clinical applications. Future work will utilise the complete dataset to improve the accuracy and reliability of these models for diagnosis.

While the preliminary dataset is limited, this work aligns with recent efforts in the field that show promise for AI-assisted diagnosis in gynaecological oncology [39,40]. Several studies have also reported improved diagnostic accuracy and decision support using AI in radiology, highlighting the potential of deep learning in medical imaging for complex classification tasks [9,41,42].

Projects such as MONAI provide specialised frameworks and a comprehensive selection of tools. This streamlines data preprocessing, model building, and evaluation, making code more accessible and optimised specifically for imaging.

Deep learning models must be rigorously validated prior to clinical deployment. Attention-based techniques like Attention MIL could be explored to refine a model’s ability to focus on the relevant regions of interest. This strategy has shown efficacy in histopathological imaging and could potentially translate well to radiology applications.

By enhancing both the model architectures and dataset scope, further studies could empower AI models to provide substantial clinical decision support, especially in pre-surgical planning and multidisciplinary team (MDT) discussions, giving healthcare professionals a powerful tool in their diagnostic arsenal.

Several limitations of this study warrant mention. Firstly, the primary focus on the methodological framework means that the full dataset has not been exhaustively analysed. As a result, potential challenges, especially those related to the quality and consistency of or anomalies in the CT and MRI scans, might remain unidentified. Secondly, the preliminary evaluations we conducted were based on a limited dataset. Given the nature of deep learning, which often benefits from vast amounts of data to achieve optimal accuracy, the outcomes obtained from this restricted dataset might not be wholly representative or generalizable.

Furthermore, the computational demands associated with implementing and deploying models like CNNs, ResNet, and DenseNet should not be understated. The true magnitude of these demands, both in terms of processing power and memory, will likely become clearer once the entire dataset is curated and subsequently subjected to rigorous model training and validation. As we integrate the full dataset and develop more complex models, concerns related to scalability and the optimisation of the training process might arise. Ensuring that these models integrate seamlessly into clinical workflows without compromising efficiency will be an additional challenge to address.

## 5. Conclusions

This study presents a preliminary framework to introduce AI into the diagnostic process for ovarian masses, focusing on using CNN, transformer, and MIL PyTorch models.

While a comprehensive analysis and validation of these models have not been undertaken, the methodological insights presented provide a foundation for planned future work in this domain. These pipelines were established and tested on a local PC workstation; however, the analysis of the complete dataset will require high-performance computing on a server equipped with NVIDIA Graphics Processing Units. The eventual objective will be to gauge the potential of these AI models for supplementing pre-surgical assessments and influencing multidisciplinary team (MDT) discussions by providing more precise imaging interpretations.

The methodologies laid out in this paper are designed to set the stage for further research, emphasizing the integration of AI and imaging techniques to potentially enhance the diagnosis of and treatment decisions for ovarian masses.

## Figures and Tables

**Table 1:** CT patients (n = 1545; median age = 64 years; range, 18–96).

	Diagnosis	n	%
Malignant (n = 922)	High-grade serous carcinoma	540	35.0
	Endometrioid carcinoma	94	6.1
	Clear cell carcinoma	75	4.9
	Mucinous carcinoma	70	4.5
	Mixed carcinoma	34	2.2
	Carcinosarcoma	26	1.7
	Unclassified malignant (NOS)	20	1.3
	Adult granulosa cell tumour	14	0.9
	Neuroendocrine carcinoma	8	0.5
	Squamous cell carcinoma	6	0.4
	Malignant teratoma	6	0.4
	Germ cell tumour, nonseminomatous	5	0.3
	Undifferentiated carcinoma	4	0.3
	Dysgerminoma	4	0.3
	Yolk sac tumour	4	0.3
	Other malignant	4	0.3
	Sertoli–Leydig cell tumour	2	0.1
	Malignant Brenner tumour	1	0.1
	Sex cord–gonadal stromal tumour, NOS	1	0.1
	Mixed germ cell tumour	1	0.1
	Leiomyosarcoma (mesenchymal)	1	0.1
	Endometrial stromal sarcoma (mesenchymal)	1	0.1
	Gastrointestinal stromal sarcoma (mesenchymal)	1	0.1
Borderline (n = 124)	Mucinous borderline tumour	63	4.1
	Serous borderline tumour	58	3.8
	Clear cell borderline tumour	1	0.1
	Endometrioid borderline tumour	1	0.1
	Gonadoblastoma	1	0.1
Benign (n = 499)	Non-neoplastic cyst	354	22.9
	Neoplasm, benign (NOS)	85	5.5
	Fibroma (sex cord–stromal)	9	0.6
	Benign teratoma (mature)	8	0.5
	Adenofibroma, NOS	7	0.5
	Adult granulosa cell tumour	7	0.5
	Mucinous cystadenoma	7	0.5
	Cystadenoma, NOS	6	0.4
	Serous cystadenoma	6	0.4
	Brenner tumour (benign)	3	0.2
	Carcinoid tumour (monodermal)	2	0.1
	Struma ovarii (monodermal)	2	0.1
	Adenoma, NOS	1	0.1
	Leiomyoma (mesenchymal)	1	0.1
	Leydig cell tumour (sex cord–stromal)	1	0.1

**Table 2:** MRI patients (n = 987; median age = 52 years; range, 18–96).

	Diagnosis	n	%
Malignant (n = 192)	High-grade serous carcinoma	63	6.4
	Endometrioid carcinoma	28	2.8
	Mucinous carcinoma	28	2.8
	Clear cell carcinoma	10	1.0
	Adult granulosa cell tumour	10	1.0
	Unclassified malignant (NOS)	9	0.9
	Mixed carcinoma	6	0.6
	Yolk sac tumour	5	0.5
	Malignant teratoma	5	0.5
	Dysgerminoma	4	0.4
	Germ cell tumour, nonseminomatous	4	0.4
	Carcinosarcoma (including MMMT)	3	0.3
	Neuroendocrine carcinoma	3	0.3
	Squamous cell carcinoma	2	0.2
	Malignant Brenner tumour	2	0.2
	Undifferentiated carcinoma	2	0.2
	Granular cell carcinoma	2	0.2
	Leiomyosarcoma (mesenchymal)	1	0.1
	Adenosarcoma (mixed epithelial–mesench.)	1	0.1
	Pseudomyxoma peritonei	1	0.1
	Gynandroblastoma (sex cord–stromal)	1	0.1
	Juvenile granulosa cell tumour	1	0.1
	Sertoli–Leydig cell tumour	1	0.1
Borderline (n = 99)	Serous borderline tumour	54	5.5
	Mucinous borderline tumour	42	4.3
	Other borderline	2	0.2
	Endometrioid borderline tumour	1	0.1
Benign (n = 696)	Non-neoplastic cyst (tumour like)	530	53.7
	Neoplasm, benign (NOS)	98	9.9
	Dermoid cyst/mature teratoma	17	1.7
	Adenofibroma, NOS	8	0.8
	Cystadenoma, NOS	8	0.8
	Serous cystadenoma	8	0.8
	Mucinous cystadenoma	7	0.7
	Fibroma (sex cord–stromal)	5	0.5
	Other benign entities	5	0.5
	Adult granulosa cell tumour	3	0.3
	Serous adenofibroma, NOS	3	0.3
	Adenoma, NOS	2	0.2
	Brenner tumour (benign)	2	0.2

**Table 3:** External CT patients from 55 centres (n = 1922; median age = 63 years; range, 18–95).

	Diagnosis	n	%
Malignant (n = 1679)	High-grade serous carcinoma	1075	55.9
	Endometrioid carcinoma	130	6.8
	Mucinous carcinoma	128	6.7
	Clear cell carcinoma	99	5.2
	Mixed carcinoma	52	2.7
	Unclassified malignant (NOS)	49	2.5
	Carcinosarcoma (including MMMT)	43	2.2
	Adult granulosa cell tumour	29	1.5
	Squamous cell carcinoma	14	0.7
	Neuroendocrine carcinoma	12	0.6
	Teratoma, malignant	9	0.5
	Undifferentiated carcinoma	6	0.3
	Dysgerminoma	6	0.3
	Pseudomyxoma peritonei	5	0.3
	Malignant Brenner tumour	4	0.2
	Mesenchymal tumours	4	0.2
	Yolk sac tumour	3	0.2
	Germ cell tumour, nonseminomatous	3	0.2
	Adenosarcoma (mixed epithelial–mesench.)	3	0.2
	Transitional cell carcinoma	2	0.1
	Lymphoma/plasmacytoma	2	0.1
	Granular cell carcinoma	1	0.1
Borderline (n = 185)	Mucinous borderline tumour	98	5.1
	Serous borderline tumour	68	3.5
	Other uncertain/borderline	6	0.3
	Sex cord–gonadal stromal tumour, NOS (borderline)	5	0.3
	Clear cell borderline tumour	4	0.2
	Borderline Brenner tumour	4	0.2
Benign (n = 58)	Neoplasm, benign (NOS)	24	1.2
	Serous cystadenoma	7	0.4
	Struma ovarii, NOS	6	0.3
	Mucinous cystadenoma	5	0.3
	Adenofibroma, NOS	4	0.2
	Cystadenoma, NOS	3	0.2
	Fibroma, NOS	2	0.1
	Sertoli cell tumour, NOS (benign)	2	0.1
	Serous adenofibroma, NOS	2	0.1
	Benign teratoma	2	0.1
	Leiomyoma	1	0.1

**Table 4:** External MRI patients from 37 centres (n = 337; median age = 55 years; range, 18–92).

	Diagnosis	n	%
Malignant (n = 163)	High-grade serous carcinoma	76	22.6
	Mucinous carcinoma	18	5.3
	Endometrioid carcinoma	16	4.7
	Clear cell carcinoma	12	3.6
	Adult granulosa cell tumour	9	2.7
	Carcinosarcoma	8	2.4
	Mixed carcinoma	5	1.5
	Unclassified malignant	5	1.5
	Squamous cell carcinoma	2	0.6
	Malignant teratoma	2	0.6
	Dysgerminoma	2	0.6
	Germ cell tumour, NOS	2	0.6
	Undifferentiated carcinoma	1	0.3
	Neuroendocrine carcinoma	1	0.3
	Gastrointestinal stromal sarcoma (mesenchymal)	1	0.3
	Yolk sac tumour	1	0.3
	Gynandroblastoma	1	0.3
	Malignant Brenner tumour	1	0.3
Borderline (n = 84)	Serous borderline tumour	35	10.4
	Mucinous borderline tumour	21	6.2
	Unclassified borderline	18	5.3
	Borderline Brenner tumour	4	1.2
	Sex cord–stromal borderline	3	0.9
	Neuroendocrine borderline	2	0.6
	Endometrioid borderline tumour	1	0.3
Benign (n = 90)	Neoplasm, benign (NOS)	45	13.4
	Benign cyst (tumour-like lesion)	10	3.0
	Fibroma (sex cord–stromal)	8	2.4
	Benign teratoma (mature)	8	2.4
	Struma ovarii (monodermal)	5	1.5
	Adenofibroma (benign epithelial)	4	1.2
	Mucinous cystadenoma	2	0.6
	Cystadenoma, NOS	2	0.6
	Sertoli cell tumour	2	0.6
	Serous adenofibroma	1	0.3
	Leiomyoma (mesenchymal)	1	0.3
	Sex cord–stromal, benign (NOS)	1	0.3
	Thecoma (sex cord–stromal)	1	0.3

**Table 5:** Internal CT and MRI scanners (n = number of scans; T = Tesla).

Modality	Manufacturer	Model	n	Field Strength (T)
CT	General Electric	Revolution HD	295	–
		LightSpeed VCT	174	–
		LightSpeed Ultra	61	–
		Revolution EVO	6	–
		Optima CT660	4	–
		Discovery 690	3	–
		HiSpeed CT/i	1	–
	Philips	Mx8000	29	–
	Siemens	Sensation 64	670	–
		SOMATOM Definition	130	–
		Sensation 16	116	–
		SOMATOM Definition AS+	21	–
		SOMATOM Force	21	–
		SOMATOM Drive	7	–
		SOMATOM AR.STAR	4	–
		SOMATOM PLUS 4	2	–
		Sensation Open	1	–
MRI	Siemens	Symphony	411	1.5
		Aera	328	1.5
		Avanto	215	1.5
		Magnetom Sola	33	1.5

**Table 6:** External CT and MRI scanners (n = number of scans; T = Tesla).

Modality	Manufacturer	Model	n	Field Strength (T)
CT	General Electric	Optima CT660	235	–
		LightSpeed VCT	81	–
		LightSpeed Ultra	19	–
		LightSpeed16	8	–
		Revolution EVO	8	–
		LightSpeed Pro 16	6	–
		Discovery 710	1	–
		Discovery CT750 HD	1	–
		LightSpeed Plus	1	–
		LightSpeed Pro 32	1	–
		Revolution HD	1	–
	Philips	Mx8000	44	–
		Brilliance 64	3	–
		Brilliance 16P	2	–
		Brilliance 40	1	–
		Ingenuity CT	1	–
	Siemens	Somatom Definition AS+	330	–
		Somatom Definition AS	223	–
		Sensation 40	196	–
		Volume Zoom	131	–
		Somatom Definition Edge	118	–
		Sensation 16	91	–
		Sensation 64	83	–
		Emotion 16	50	–
		Sensation 4	41	–
		Emotion 6	10	–
		Somatom go.All	7	–
		Spirit	4	–
		SOMATOM Definition Flash	1	–
		Somatom go.Top	1	–
		Symbia T16	1	–
	Toshiba	Aquilion	145	–
		Aquilion ONE	31	–
		Aquilion Prime SP	28	–
		Aquilion PRIME	16	–
		Asteion	2	–
MRI	General Electric	Signa HDxt	3	1.5
		Optima MR450w	3	1.5
		Discovery MR450	3	1.5
		Signa HDx	1	1.5
	Philips	Intera	48	1.5
		Ingenia	34	1.5
		NT Intera	30	1.5
		Achieva	27	1.5
	Siemens	Aera	99	1.5
		Avanto	51	1.5
		Avanto_fit	26	1.5
		SymphonyTim	6	1.5
		Symphony	3	1.5
		Magnetom Sola	2	1.5
		Amira	1	1.5

## Data Availability

The data presented in this study are available on request from the corresponding author. The data are not publicly available due to institutional data-sharing restrictions and cannot be shared publicly without additional approvals from the relevant institutional committees.

## Supplementary Materials

The following supporting information can be downloaded at: www.mdpi.com/article/10.3390/jpm15020076/s1, Figure S1: Flowchart outlining the diagnostic and management pathways for suspected malignant pelvic masses, showing how imaging findings influence the timing of tissue biopsy and surgical intervention; Table S1: Data augmentation steps; Table S2: Software tools, versions, and references, listing the primary software tools and libraries used throughout this study, including their version references and principal functions; Table S3: A layer-by-layer breakdown and comparative summary of four different 3D neural network architectures—3D ResNet-50, 3D DenseNet-121, a typical 3D U-Net (UNest), and an MIL Attention Model; Table S4: Hyperparameter search space.

## Abbreviations

The following abbreviations are used in this manuscript:

2D	Two dimensional
3D	Three dimensional
ADAM	Adaptive Moment Estimation
AI	Artificial intelligence
AvgPool	Global average pooling
BN	Batch normalisation
CNN	Convolutional neural network
ComBat	Combating batch effects when combining batches
Conv	Convolution
CSV	Comma-separated values
CT	Computed tomography
dec	Decoder
DenseNet	Dense convolutional network
DICOM	Digital Imaging and Communications in Medicine
DL	Deep learning
DWI	Diffusion-weighted imaging
enc	Encoder
EnsureTyped	Ensure Type (dictionary-based) Transform
FC	Fully connected
FN	False Negative
FP	False Positive
GPU	Graphics processing unit
Grad-CAM	Gradient-weighted Class Activation Mapping
IRAS	Integrated Research Application System
JSON	JavaScript Object Notation
Matplotlib	A Python library for data visualisation
MaxPooling3D	Three-dimensional max-pooling layer
MDT	Multidisciplinary team
MIL	Multiple-Instance Learning
ML	Machine learning
MLP	Multi-Layer Perceptron
MONAI	Medical Open Network for Artificial Intelligence
MRI	Magnetic resonance imaging
NesT	Nested transformer
NIfTI	Neuroimaging Informatics Technology Initiative
NOS	Not otherwise specified
NumPy	A Python library for numerical computations
OC	Ovarian cancer
PACS	Picture Archiving and Communication System
pt	PyTorch tensor file format
PyTorch	A Python-based deep learning framework
Rand3DElasticd	Random 3D elastic (dictionary-based) transform
RandAdjustContrastd	Random adjust contrast (dictionary-based) transform
RandAffined	Random affine (dictionary-based) transform
RandFlipd	Random flip (dictionary-based) transform
RandGaussianNoised	Random Gaussian noise (dictionary-based) transform
RandZoomd	Random zoom (dictionary-based) transform
RCT	Randomised controlled trial
ReLU	Rectified Linear Unit
ResNet	Residual Network
ROI	Region of interest
T	Tesla
Tanh	Hyperbolic tangent activation function
TCGA-OV	The Cancer Genome Atlas Ovarian dataset
TN	True Negative
TP	True Positive
UNeST	Universal network with nested transformer
UNet	U-shaped encoder–decoder network architecture
