# Supplementary material for: A Methodological Framework for AI-Assisted Diagnosis of Ovarian Masses Using CT and MR Imaging
Source: J Pers Med. 2025 Feb 19;15(2):76. doi: 10.3390/jpm15020076 (PMC11856859; doi:10.3390/jpm15020076)
Supplement: Supplementary file 1 [file jpm-15-00076-s001.zip › jpm-3467484-supplementary.pdf]

Supplemental Table S1: Data Augmentation Steps

Transformation	Parameters / Probability	Description / Effect
**RandFlipd**	spatial_axis=[0, 1, 2], prob=0.5	Randomly flips the image along the x, y, or z axes with 50% probability. Improves rotational/orientation invariance by mirroring anatomical structures.
**RandAffined**	rotate_range=(0.1, 0.1, 0.1), prob=0.5	Applies small random rotations (±0.1 radians) around each axis with 50% probability. Increases robustness to variations in patient positioning.
**RandGaussianNoised**	prob=0.2	Adds Gaussian noise with 20% probability. Helps the model learn to handle scanner or acquisition noise.
**RandZoomd**	min_zoom=0.9, max_zoom=1.1, prob=0.3	Randomly zooms in or out by 10% with 30% probability. Simulates variations in scale and field of view.
**RandAdjustContrastd**	gamma=(0.5, 1.5), prob=0.2	Random gamma-based contrast adjustments with 20% probability. Addresses differences in tissue contrast.
**Rand3DElasticd**	sigma_range=(5,10), magnitude_range=(1,2), prob=0.3	Deforms the image elastically with 30% probability to mimic realistic anatomical variations or distortions.
**EnsureTyped**	(Always applied)	Ensures the augmented data is converted to a PyTorch tensor on the CPU, ready for downstream deep learning tasks

Supplemental Table S2: Software Tools, Versions, and References. Primary software tools and libraries used throughout the study, including their version references, principal functions

Software / Library	Description / Usage	Link / Reference
**dcm2niix**	Converts DICOM to NIfTI format, preserving key metadata.	https://github.com/rordenlab/dcm2niix
**NiBabel**	Python library to read/write neuroimaging data (NIfTI).	https://nipy.org/nibabel/gettingstarted.html
**MONAI**	PyTorch-based framework for deep learning in medical imaging.	https://monai.io/
**Optuna**	Hyperparameter optimization framework with various samplers.	https://optuna.org/
**PyTorch**	Primary deep learning framework for building neural networks.	https://pytorch.org/
**TensorBoard**	Visualization tool for training metrics, losses, graphs, etc.	https://www.tensorflow.org/tensorboard
**itk-Snap**	Interactive tool for manual segmentation / ROI annotation.	http://www.itksnap.org/

Supplemental Table S3: A layer-by-layer breakdown and comparative summary of four different 3D neural network architectures—3D ResNet-50, 3D DenseNet-121, a typical 3D U-Net (UNest), and an MIL Attention model.

Aspect	3D ResNet-50	3D DenseNet-121	UNest (Typical 3D U-Net)	MIL Attention
**Layer-by-Layer Breakdown**	• Stem / Initial:<br>- Conv1 (7×7×7 kernel, stride=2)<br>- BN → ReLU → MaxPool (3D)<br>• Main Residual Layers:<br>- Layer1: 3 "bottleneck" blocks<br>- Layer2: 4 "bottleneck" blocks<br>- Layer3: 6 "bottleneck" blocks<br>- Layer4: 3 "bottleneck" blocks<br>• Each bottleneck typically has 3 Conv layers (1×1→3×3→1×1), all in 3D.<br>Finally, an avg-pool + FC for classification.	• Stem / Initial:<br>- Conv1 (7×7×7 kernel, stride=2)<br>- BN → ReLU → MaxPool (3D)<br>• Four Dense Blocks (DB1–DB4):<br>Each consists of multiple "dense layers." Each "dense layer" typically has:<br>○ BN → ReLU → 1×1×1 conv<br>○ BN → ReLU → 3×3×3 conv<br>Between dense blocks are transition layers that include 1×1×1 conv + 3D avg-pool.<br>• Ends with an avg-pool + FC for final class scores.	• Encoder (Down):<br>Typically 4 or 5 levels; each level has 2 (sometimes 3) consecutive 3D conv layers + BN/ReLU.<br>Then a 3D pool (or strided conv) reduces spatial size.<br>• Bottleneck / Bottom:<br>2 or 3 more 3D conv layers.<br>• Decoder (Up):<br>4 or 5 levels mirrored from encoder; each level has up-sampling (or transposed conv) + skip connection + 2 (or 3) 3D conv layers.<br>Final layer outputs num_classes feature maps.	• Same Backbone as 3D ResNet-50 (Conv1, BN, ReLU, MaxPool, 4 residual layers of bottleneck blocks).<br>• Attention Block inserted after the final spatial feature map:<br>○ MLP #1: Linear(2048→128) + Tanh<br>○ MLP #2: Linear(128→1)<br>○ Softmax along spatial dimension to get patch weights.<br>Therefore, the 3D convolutional portion is identical to 3D ResNet-50, and the attention portion uses only linear layers.
**Number of Conv Layers per Block**	- Each bottleneck block: 3 conv layers (1×1, then 3×3, then 1×1).<br>- Layer1: 3 blocks × 3 convs/block = 9 conv layers total.<br>- Layer2: 4 blocks = 12 convs,<br>- Layer3: 6 blocks = 18 convs,<br>- Layer4: 3 blocks = 9 convs.	- Dense Block example:<br>DB1: 6 "dense layers" × (2 convs/layer) = 12<br>DB2: 12 "dense layers" = 24 convs<br>DB3: 24 "dense layers" = 48 convs	- Encoder level: Typically 2 or 3 consecutive 3D conv layers, repeated for ~4–5 levels.<br>- Bottleneck: ~2–3 convs.<br>- Decoder level: Another 2 or 3 conv layers per level for 4–5 levels.	- Per "bottleneck block" in the backbone: same as 3D ResNet-50 (3 convs per block).<br>- The attention block does not add new 3D convs; only linear layers.

		DB4: 16 "dense layers" = 32 convs<br>- Transition layer has 1×1×1 conv.<br>- Summation yields total conv count (plus the initial conv).	Total ~20–30 conv layers, depending on the design.	
**Approx. Total Conv Count**	- Initial Conv1 = 1<br>- L1–L4 = 9+12+18+9 = 48 convs<br>- Overall: ~49–53 total 3D conv layers (depending on downsample ops inside blocks).	- DB1 (12) + DB2 (24) + DB3 (48) + DB4 (32) = 116 from dense blocks<br>- + ~3 from transitions & initial conv = ~120–125 total.	- Typically ~20–30 total 3D conv layers for standard U-Net.<br>- Extended versions can go 40+ layers if deeper expansions are used.	- Matches ~49–53 conv layers (3D ResNet-50 backbone).<br>- Attention MLP adds no new 3D convs.
**Notes / Unique Details**	- Uses 3D "bottleneck" blocks to keep parameter counts manageable.<br>- Ends with a global avg-pool + FC→softmax.	- Dense connections feed each layer's output to subsequent layers.<br>- Transition layers reduce channels and spatial dims.<br>- Ends with global avg-pool + FC→softmax.	- Primarily for segmentation: final output is num_classeschannels for voxel-wise predictions.<br>- Skip connections help retain resolution from earlier layers.<br>- Deeper or shallower variants exist.	- Same as 3D ResNet-50, plus an attention mechanism for MIL.<br>- Ideal for focusing on key 3D regions (tumors, lesions, etc.).

Supplementary Table S4: Hyperparameter Search Space

Hyperparameter	Search Method	Range/Options
**batch_size**	suggest_categorical	[4, 8, 16, 32]
**lr** (learning rate)	suggest_float (log)	1e-5 to 3e-4
**weight_decay**	suggest_float (log)	1e-7 to 1e-4
**gamma**	suggest_float	0.0 to 2.5
**alpha_factor**	suggest_float	0.5 to 2.0
**minority_acc_threshold**	suggest_float	0.1 to 0.7
**minority_boost_factor**	suggest_float	1.2 to 1.5

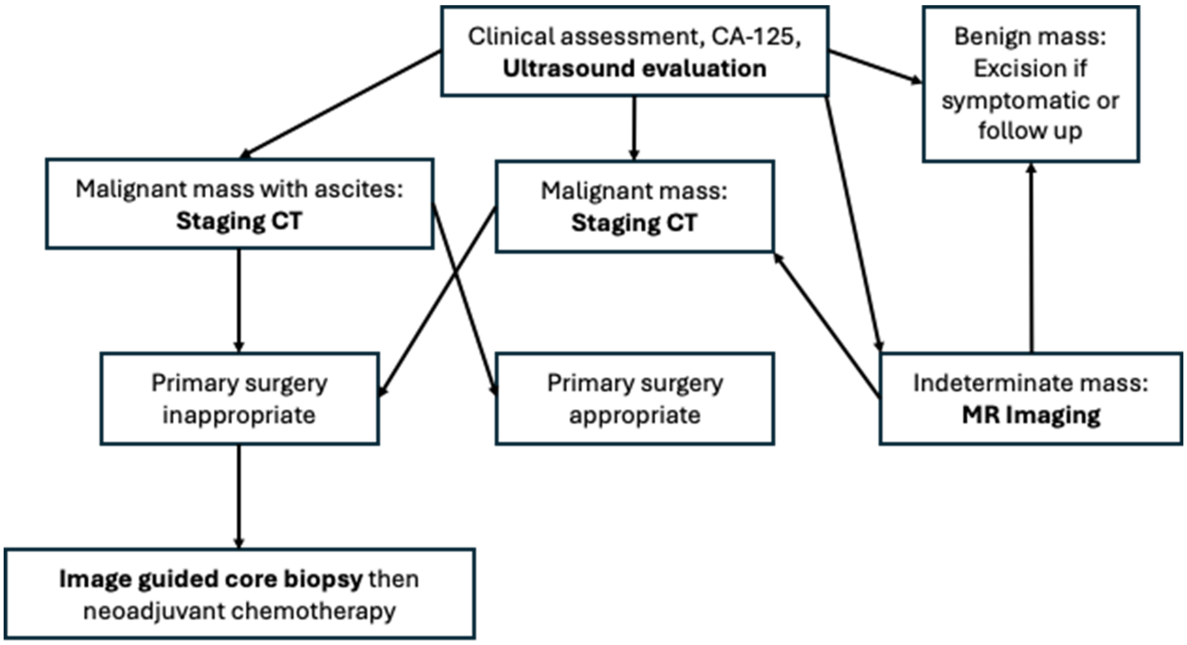

Supplemental Figure S1: Flowchart outlining the diagnostic and management pathways for suspected malignant pelvic masses, showing how imaging findings influence the timing of tissue biopsy and surgical intervention.

Adopted from Swift, S.E., Weston, M.J., Mohamed, M.B., Scarsbrook, A., Wilkinson, N. (2023). Integration of Imaging and Pathology in the Multidisciplinary Process. In: Wilkinson, N. (eds) Pathology of the Ovary, Fallopian Tube and Peritoneum. Essentials of Diagnostic Gynecological Pathology. Springer, Cham. https://doi.org/10.1007/978-3-031-39659-5_6
